# The Role of Carbenicillin as an Inhibitor of the Biofilm Regulator CsgD in *Salmonella* Typhimurium

**DOI:** 10.1002/mbo3.70081

**Published:** 2025-10-21

**Authors:** Negar Narimisa, Amin Khoshbayan, Faramarz Masjedian Jazi, Shabnam Razavi

**Affiliations:** ^1^ Microbial Biotechnology Research Center Iran University of Medical Sciences Tehran Iran; ^2^ Department of Microbiology School of Medicine, Iran University of Medical Sciences Tehran Iran

**Keywords:** biofilm, carbenicillin, CsgD, molecular docking, *Salmonella* Typhimurium

## Abstract

*Salmonella* Typhimurium, a major foodborne pathogen, forms biofilms that enhance its environmental persistence and resistance to antibiotics, presenting significant public health challenges. The CsgD protein, a key transcriptional regulator, orchestrates biofilm formation by regulating curli fimbriae and cellulose production. This study aimed to identify and evaluate potential CsgD inhibitors to disrupt *S.* Typhimurium biofilms using a combination of computational and experimental methodologies. Molecular docking was performed to screen 145 FDA‐approved antibiotics from DrugBank against the CsgD protein. Carbenicillin, identified as a top candidate, was further analyzed through 100 ns molecular dynamics simulations to assess the stability of the carbenicillin‐CsgD complex. Experimental evaluations determined the minimum biofilm inhibitory concentration (MBIC), and minimum biofilm eradication concentration (MBEC) of carbenicillin against *S.* Typhimurium isolates. Biofilm structure and curli production were examined using scanning electron microscopy (SEM) and Congo red agar assays, respectively. Molecular docking revealed carbenicillin's high binding affinity to CsgD. Molecular dynamics simulations confirmed the structural stability of the carbenicillin‐CsgD complex. Experimental assays established MBIC and MBEC at 1 and 4 μg/mL, respectively. SEM analysis showed morphological changes and disrupted biofilm architecture at 0.5–1 μg/mL carbenicillin, while Congo red agar assays demonstrated dose‐dependent suppression of curli production. Carbenicillin exhibits significant potential as a CsgD‐targeted anti‐biofilm agent, providing a foundation for novel therapeutic strategies to combat *S.* Typhimurium infections and address their public health burden.

## Graphical abstract

*Salmonella* Typhimurium biofilms, driven by the CsgD protein, enhance antibiotic resistance and pose public health concerns. This study employed molecular docking to screen 145 FDA‐approved antibiotics, identifying carbenicillin as a potent CsgD inhibitor. Molecular dynamics simulations confirmed stable carbenicillin‐CsgD binding. Experimental assays revealed a minimum biofilm inhibitory concentration (MBIC) of 1 μg/mL and minimum biofilm eradication concentration (MBEC) of 4 μg/mL. Scanning electron microscopy and Congo red agar assays demonstrated disrupted biofilm architecture and reduced curli production. Carbenicillin's anti‐biofilm efficacy highlights its potential as a novel therapeutic to combat *S*. Typhimurium infections.

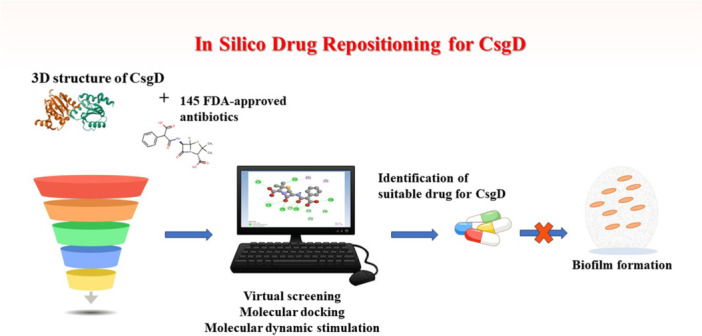

## 1 Introduction

Salmonellosis, primarily caused by *Salmonella* subspecies *enterica*, represents a significant global public health challenge due to its widespread impact as a foodborne illness (Griffith et al. 2019). This pathogen affects a diverse range of hosts, including mammals, birds, fish, and humans, contributing to substantial morbidity and economic losses (Narimisa et al. 2021). With approximately 2600 serotypes, *Salmonella* exhibits remarkable genetic diversity, enabling adaptation to varied environments and hosts (Deng et al. 2025).

Most human infections are attributed to a limited number of serotypes, broadly categorized as typhoidal (e.g., serovars Typhi and Paratyphi A) or non‐typhoidal (Narimisa et al. 2025). Among non‐typhoidal *Salmonella* (NTS) serotypes, *Salmonella* Typhimurium stands out as one of the most prevalent, capable of causing gastroenteritis and systemic infections in humans and animals (Narimisa et al. 2024a).

Biofilms, structured communities of bacteria encased in a self‐produced matrix of polysaccharides, proteins, and extracellular DNA, confer significant survival advantages (Dsouza et al. 2024). These matrices facilitate bacterial adhesion to surfaces, shield against host immune responses, and enhance resistance to environmental stressors, including antimicrobial agents (Dsouza et al. 2024). In *S*. Typhimurium, biofilm formation on biotic and abiotic surfaces, such as food processing equipment and medical devices, amplifies its environmental persistence and pathogenicity (Obe et al. 2022). Biofilm‐associated bacteria demonstrate up to 1000‐fold greater resistance to antibiotics compared to planktonic cells, a phenomenon driven by the protective extracellular matrix, reduced metabolic activity, and the presence of persister cells (Venkatesan et al. 2015). This heightened resistance poses significant challenges to treating *S*. Typhimurium infections, necessitating innovative approaches to target biofilm formation (Venkatesan et al. 2015).

The biofilm matrix of *S*. Typhimurium comprises key components, including cellulose, proteins, and curli fimbriae (Yadav et al. 2025). Curli, amyloid‐like protein structures, promote intercellular adhesion and surface attachment, while cellulose provides structural stability to the matrix (Tursi and Tükel 2018). The production of these components is tightly regulated by the CsgD protein, a transcriptional regulator encoded by the *csgD* gene within the curli operon (*csgDEFG*) (Khambhati et al. 2021). The CsgD protein activates the *csgBAC* operon, which encodes curli fimbriae subunits, and indirectly regulates cellulose synthesis through the *adrA* gene (Liu et al. 2014). Mutations disrupting *csgD* abolish curli and cellulose production (Brombacher et al. 2006), severely impairing biofilm formation and bacterial persistence, underscoring CsgD's critical role in biofilm biogenesis.

The identification of novel bioactive molecules is a resource‐intensive and time‐consuming process. Drug repurposing, which involves identifying new therapeutic applications for existing drugs, offers a cost‐effective and efficient alternative for addressing these challenges (Ng et al. 2021; Rudrapal et al. 2020). In this context, targeting CsgD presents a promising strategy for disrupting biofilm integrity and enhancing the efficacy of antimicrobial treatments. By inhibiting CsgD's regulatory function, it may be possible to compromise the structural and functional stability of *S*. Typhimurium biofilms, rendering bacteria more susceptible to antibiotics and host defenses.

This study seeks to assess the efficacy of FDA‐approved antibiotics in targeting the CsgD protein through computational approaches, including molecular docking and dynamics simulations, to identify the most potent inhibitor of CsgD function and evaluate its effects on biofilm formation. These findings enhance our understanding of biofilm regulation in *S*. Typhimurium and provide valuable insights into novel strategies for reducing the public health impact of this pathogen.

## 2 Materials and Methods

### 2.1 Protein‐Ligand Preparation

The X‐ray crystallographic structures of CsgD (PDB ID: 5XP0) were retrieved from the RCSB Protein Data Bank with a resolution of 2.0 Å (Rose et al. 2016). These structures were processed through preparation steps, including the addition of hydrogen atoms, removal of water molecules, and energy minimization, utilizing UCSF Chimera version 1.15 software (Meng et al. 2006).

A library of FDA‐approved antibiotics, comprising 145 compounds, was obtained from DrugBank database (Wishart 2006). These antibiotics were screened, and their structures were downloaded in SDF format. The ligands were then optimized and converted into PDBQT format using the PyRx virtual screening tool's graphical user interface (Dallakyan and Olson 2014).

### 2.2 Molecular Docking and Virtual Screening

The PyRx software, utilizing AutoDock VINA as its docking engine, was employed for the molecular screening of all ligands (Pawar and Rohane 2021). During the docking process, ligands were treated as flexible, while the protein was kept rigid (Pawar and Rohane 2021). Grid parameter configuration files were created using the AutoGrid engine within PyRx version 0.8. The selected molecules were docked into the active site of CsgD, guided by literature reference (Wen et al. 2017). Default docking parameters were used to generate 10 docked conformations for each compound (Nguyen and Wei 2019). The ligand exhibiting the highest (most negative) binding energy was deemed to have the greatest binding affinity (Nguyen and Wei 2019). Upon completion of docking, multiple factors, including binding energy and hydrogen bond interactions, were evaluated. After a thorough analysis of ligand‐receptor interactions, the resulting complexes were saved and visualized using UCSF Chimera, Discovery Studio 3.5, and the Protein‐Ligand Interaction Profiler (PLIP).

### 2.3 Molecular Dynamics (MD) Simulation

MD simulations were performed on both the unbound protein and the docked complex using GROMACS 2022.6 software with the CHARMM27 force field (Bauer et al. 2023). The protein and protein‐ligand complex were solvated in a cubic box containing TIP3P water molecules. To ensure a physiologically relevant ionic strength of 0.15 M and to neutralize the system's charge, suitable amounts of Na^+^ and Cl^−^ ions were incorporated. Energy minimization was conducted over 50,000 steps using the steepest descent algorithm for each simulation (Meza 2010). The simulations were run at 300 K, following a two‐stage equilibration process: NVT and NPT, each lasting 100 ps at 300 K. Temperature and pressure were maintained using the Berendsen thermostat (Berendsen et al. 1987), and the Parrinello‐Rahman barostat (Parrinello and Rahman 1981), respectively. Subsequently, 100 ns MD simulations were carried out with a 2‐femtosecond time step. The LINCS algorithm (Hess et al. 1997) was utilized to calculate van der Waals interactions, long‐range electrostatics, and covalent bond constraints. The resulting MD trajectories were analyzed using GROMACS tools to extract relevant data insights.

### 2.4 Bacterial Strain and Growth Conditions

In this study, *S*. Typhimurium ATCC 14028 and a clinical isolate sourced from the Microbiology Department of Iran University of Medical Sciences were used. Both isolates were preserved at −80°C in Brain Heart Infusion (BHI) broth containing 20% glycerol.

### 2.5 Determination of Minimum Inhibitory Concentration (MIC) and Minimum Bactericidal Concentration (MBC)

The MIC and MBC of carbenicillin against S. Typhimurium ATCC 14028 and a clinical isolate were determined using the broth microdilution method in 96‐well U‐bottom plates (Wiegand et al. 2008; Koeth et al. 2015). Briefly, 100 μL of Mueller‐Hinton Broth (MHB) was dispensed into each well, followed by 100 μL of carbenicillin solution in the first well and serial twofold dilutions. Each well was inoculated with 100 μL of an overnight bacterial culture (~10^5 cells) and incubated at 37°C for 24 h. The MIC was the lowest concentration preventing visible growth. For MBC, 5 μL from wells without visible growth were plated on MH agar, with MBC defined as the lowest concentration showing no colony growth after 24 h at 37°C. All experiments were conducted in triplicate.

### 2.6 Determination of Minimum Biofilm Inhibitory Concentration (MBIC) and Minimum Biofilm Eradication Concentration (MBEC)

In this study, a clinical isolate of *S*. Typhimurium previously characterized as a strong biofilm producer (Narimisa et al. 2024a), along with the standard strong biofilm‐forming strain *S*. Typhimurium ATCC 14028, was used to assess the effect of carbenicillin on biofilm development.

The MBIC of carbenicillin was determined using 96‐well polystyrene microtiter plates with LB broth (no sodium chloride) supplemented with varying carbenicillin concentrations (Thieme et al. 2019). Plates were incubated at 30°C for 24 h to allow biofilm formation. Post‐incubation, wells were washed with PBS, air‐dried, fixed with 200 μL methanol for 20 min, and stained with 1% crystal violet for 15 min. After washing with distilled water and air‐drying, bound stain was solubilized with 200 μL 95% ethanol. Biofilm biomass was quantified at 570 nm using an ELISA reader (Thermo Fisher Scientific, USA). LB medium (no sodium chloride) served as the negative control, and LB with bacteria but no antibiotic as the positive control. The MBIC was defined as the lowest concentration reducing biofilm biomass to levels statistically indistinguishable from the negative control. All experiments were performed in triplicate.

To evaluate the MBEC, planktonic cells were removed after biofilm formation, and the wells were washed twice with PBS. Then, 200 μL of LB broth without sodium chloride containing different concentrations of carbenicillin was added to each well, followed by incubation at 30°C for another 24 h. Viable biofilm cells were recovered by gently scraping the well surface with a sterile plastic pipette tip, and bacterial counts were determined by serial dilution and plating onto Tryptic Soy Agar (TSA), followed by incubation at 37°C for 24 h. For each strain, the MBEC was defined as the lowest antibiotic concentration that resulted in at least a six‐log reduction in viable biofilm cells compared to the untreated control (Kolypetri et al. 2023). All experiments were conducted in triplicate.

### 2.7 Light Microscopy Analysis

To investigate the impact of carbenicillin on biofilm formation by *S*. Typhimurium ATCC 14028 and a clinical isolate, biofilms were formed on glass coverslips placed in 6‐well plates. Each coverslip was inoculated with a bacterial suspension (10⁸ CFU/mL in LB medium) containing either 0.5 or 1 μg/mL carbenicillin. Following 24 h of incubation at 30°C, the supernatant was removed, and the wells were gently washed twice with PBS. The coverslips were then collected, stained with 1% crystal violet for 20 min, and rinsed three times with 200 μL of distilled water to remove excess stain. After air‐drying, the biofilm structures were examined using a light microscope (BX53, Olympus, Tokyo, Japan) (Yang et al. 2019; Adnan et al. 2020).

### 2.8 Scanning Electron Microscopy Analysis

The effect of carbenicillin on biofilm formation was further evaluated using scanning electron microscopy (SEM), following a previously reported protocol (Narimisa et al. 2024a). Bacterial suspensions of *S*. Typhimurium ATCC 14028 and a clinical isolate (10⁸ CFU/mL) were prepared in LB broth supplemented with either 0.5 or 1 μg/mL carbenicillin. These suspensions were incubated without agitation at 30°C for 24 h on glass coverslips placed in 6‐well polystyrene plates. Post‐incubation, the media were discarded, and each well was washed twice with sterile distilled water. The samples were fixed in 2.5% glutaraldehyde for 4 h, followed by three rinses with sterile distilled water. Subsequently, the cells were dehydrated using a graded ethanol series (30% to 100%). The coverslips were air‐dried at room temperature, sputter‐coated with gold, and analyzed under a scanning electron microscope (AIS2100, Seron Technology).

### 2.9 Congo Red Assay

A 5 μL aliquot of an overnight culture of *S*. Typhimurium isolates was spotted onto the center of Congo Red LB agar (without NaCl, supplemented with 40 μg/mL Congo Red) for the control group, or Congo Red LB agar (without NaCl, supplemented with 40 μg/mL Congo Red and 0.5 or 1 μg/mL carbenicillin) for the treated group, and incubated at 30°C for 72 h to evaluate colony morphology (Li et al. 2024).

### 2.10 Statistical Analysis

Statistical analyses were performed using GraphPad Prism 8 (GraphPad Software Inc.). One‐way analysis of variance (ANOVA) was conducted, followed by Tukey's post‐hoc test, with statistical significance defined at *p* < 0.05.

## 3 Results

### 3.1 Molecular Docking and Interaction Analysis

The docking study employed FDA‐approved antibiotics as ligands against the CsgD protein of *S*. Typhimurium, providing comprehensive insights into molecular interactions. A total of 145 antibiotics were initially obtained from DrugBank. Twelve distinct drugs exhibiting binding affinities of −6.5 kcal/mol or higher for the CsgD protein were determined. The binding affinities and names of the top seven ligands are summarized in Table 1. However, several top‐ranked drugs failed to interact with critical amino acid residues in CsgD's active site, such as ASP 59 and ASN 88. Among the evaluated compounds, carbenicillin demonstrated the highest binding efficacy, achieving a docking score of −7.4 kcal/mol. Analysis using PLIP confirmed hydrogen bond formation between carbenicillin and key residues within CsgD's active site.

**Table 1:** Docking scores of top antibiotics following virtual screening.

No	DrugBank ID	Name	Binding affinity (KCal/mol)	Key residues interaction
1	DB00578	Carbenicillin	−7.4	ASP 59 and ASN 88
2	DB01060	Amoxicillin	−7.2	ASN 88
3	DB01053	Benzylpenicillin	−7.1	No H‐bond interaction
4	DB00417	Phenoxymethylpenicillin	−7.1	No H‐bond interaction
5	DB13337	Pheneticillin	−6.8	No H‐bond interaction
6	DB01607	Ticarcillin	−6.8	No H‐bond interaction
7	DB00607	Nafcillin	−6.7	No H‐bond interaction

**Figure 1:**
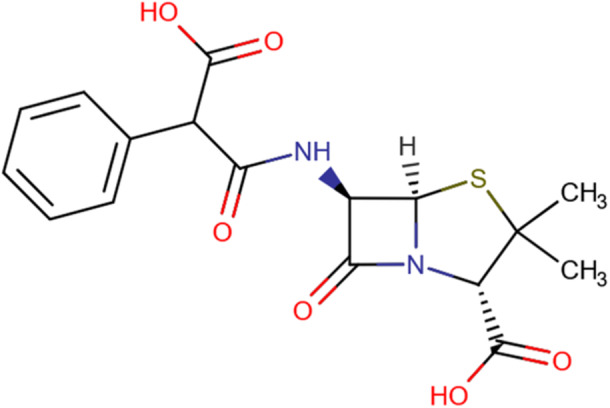
Two‐dimensional illustration of Carbenicillin's structure.

**Figure 2:**
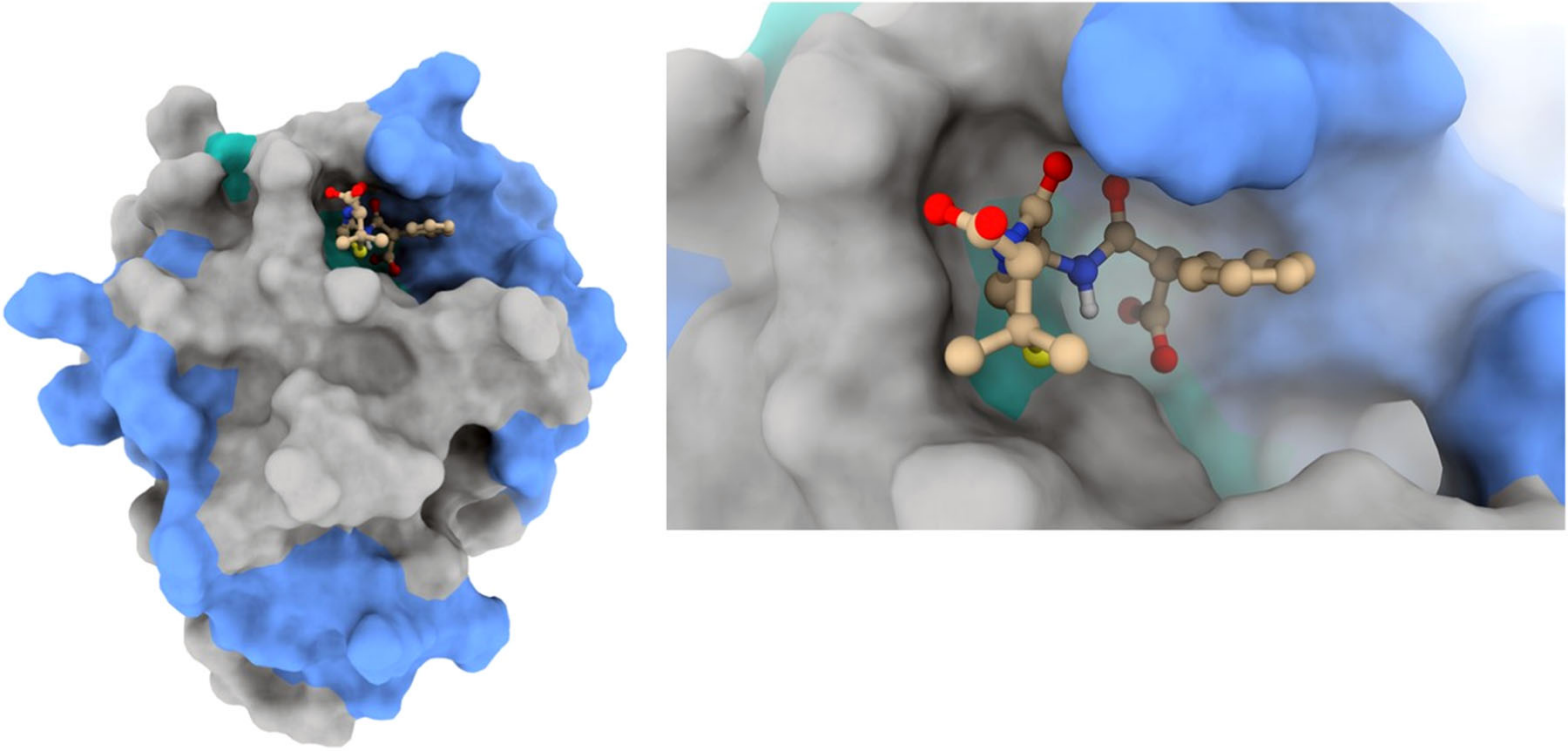
Molecular docking view of Carbenicillin within the binding site of CsgD.

**Figure 3:**
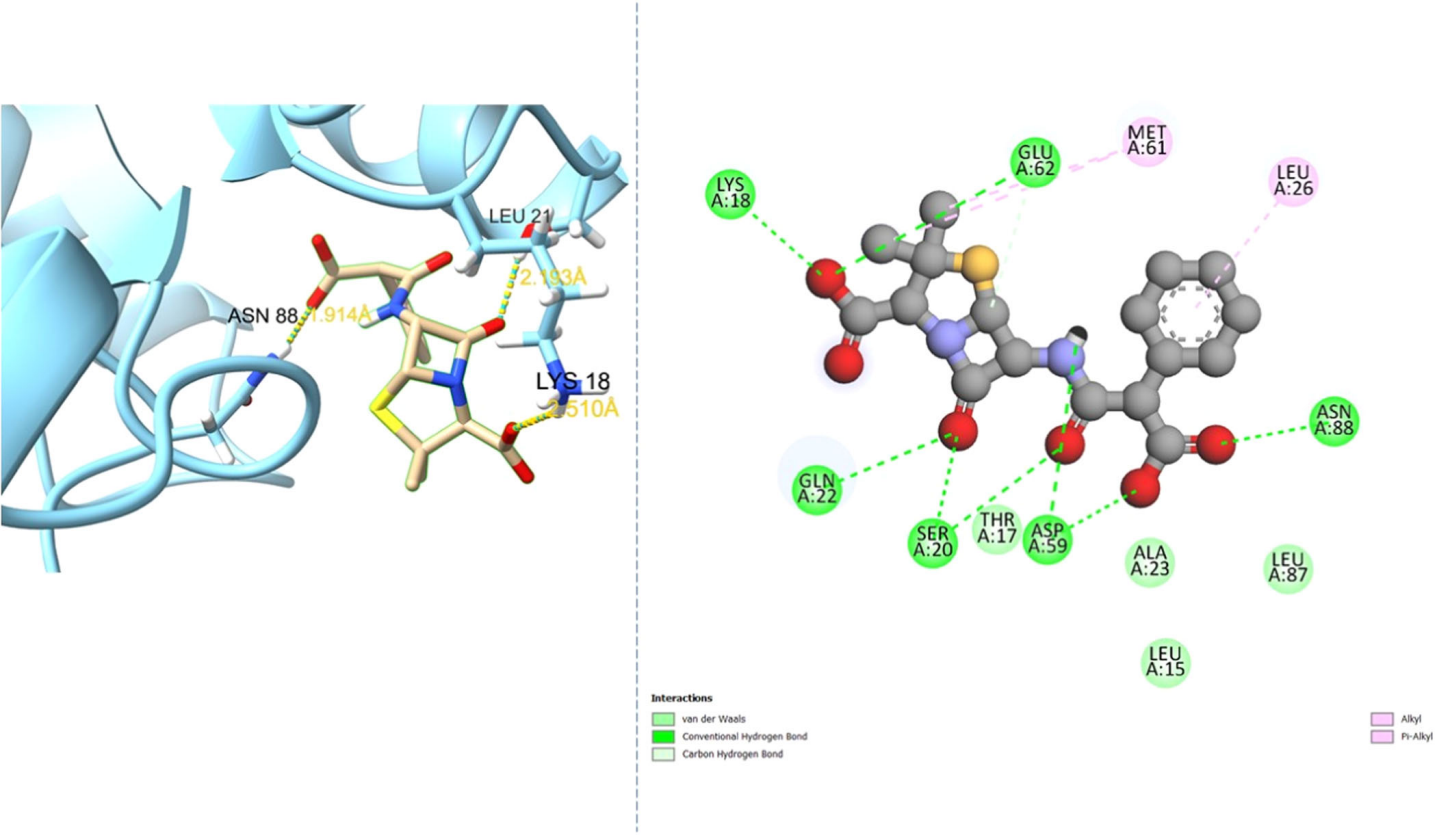
Hydrogen bond interactions from the docking analysis of Carbenicillin in the CsgD binding pocket.

A two‐dimensional depiction of carbenicillin's molecular structure is presented in Figure 1. Visualizations of carbenicillin's docking patterns and hydrogen bond interactions, generated using UCSF Chimera and Discovery Studio, are shown in Figures 2 and 3, respectively.

### 3.2 MD Simulation

Based on the docking results, carbenicillin, identified as the most effective ligand for the CsgD protein, was selected for further MD simulation studies. MD simulations are a powerful computational approach for elucidating protein‐ligand interactions, providing detailed insights into the spatial orientation of ligands at the protein's active site, conformational dynamics, and molecular stability (Grewal et al. 2025).

**Figure 4:**
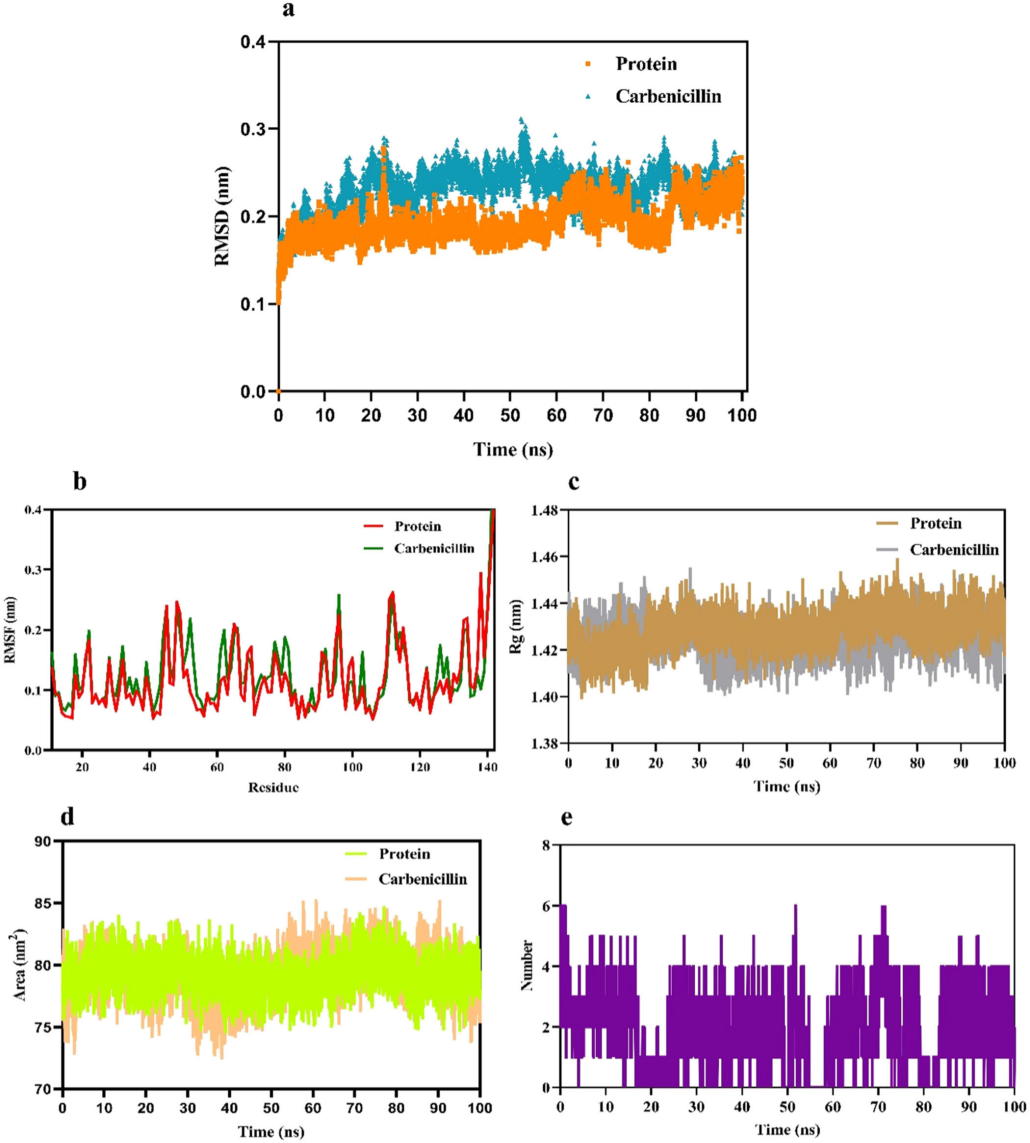
(a) RMSD graphs of the backbone atoms of CsgD in its free state and with Carbenicillin. (b) RMSF graphs of CsgD in its free state and with Carbenicillin. (c) Rg graphs of CsgD in its free state and with Carbenicillin. (d) SASA graphs of CsgD in its free state and with Carbenicillin. (e) Count of hydrogen bonds formed between CsgD atoms and Carbenicillin.

All‐atom MD simulations were conducted on the carbenicillin‐CsgD protein complex for 100 ns at 300 K. The stability and dynamics of the complex were evaluated using Root Mean Square Deviation (RMSD) and Root Mean Square Fluctuation (RMSF) analyses. The RMSD analysis offered valuable insights into the protein's structural dynamics, verifying its stability and equilibrium throughout the simulation period (Khan et al. 2021). The RMSD results for the carbenicillin‐CsgD complex indicated structural stability throughout the 100 ns simulation (Figure 4a). The average RMSD values were 0.193 for unbound CsgD and 0.230 for the carbenicillin‐CsgD complex, reflecting a slight increase in RMSD in the presence of carbenicillin. These findings suggest that carbenicillin binding does not compromise the structural integrity of the enzyme, as evidenced by the stable RMSD values observed during the simulation.

The RMSF analysis, depicted in Figure 4b, was used to evaluate residue fluctuations and the dynamic stability of the system over the 100 ns simulation (Priya et al. 2015). The RMSF values for unbound CsgD and the carbenicillin‐CsgD complex were 0.118 and 0.127, respectively, indicating sustained stability in both systems throughout the simulation period.

To further assess the compactness of the protein, the radius of gyration (Rg) was calculated for both unbound CsgD and the carbenicillin‐CsgD complex (Figure 4c). The Rg values were 1.42 for both systems, showing no significant variation in the Rg trajectory and confirming the structural stability of the complex.

Additionally, the solvent‐accessible surface area (SASA) of the protein‐ligand complex was analyzed to evaluate hydrophobic interactions contributing to conformational stability in the solvent environment (Zhang et al. 2021). The SASA values for unbound CsgD and the carbenicillin‐CsgD complex were 79.17 and 79.13 nm², respectively (Figure 4d). These results indicate minimal changes in solvent accessibility, supporting the stable conformational dynamics of the ligand‐bound structure.

Hydrogen bond interactions, critical for stabilizing the ligand within the protein's active site (Chen et al. 2016), were also analyzed. The evolution of hydrogen bond interactions with carbenicillin is shown in Figure 4e, revealing a maximum of six hydrogen bonds between CsgD and carbenicillin. The analysis confirmed stable and effective hydrogen bond interactions, with carbenicillin remaining securely bound within the active site throughout the simulation.

### 3.3 Determination of MIC and MBC

The MIC and MBC of carbenicillin were evaluated for *S*. Typhimurium ATCC 14028 and a clinical isolate using a broth microdilution assay. The MIC and MBC values for both isolates were determined to be 8 and 32 μg/mL, respectively.

### 3.4 Determination of MBIC and MBEC

**Figure 5:**
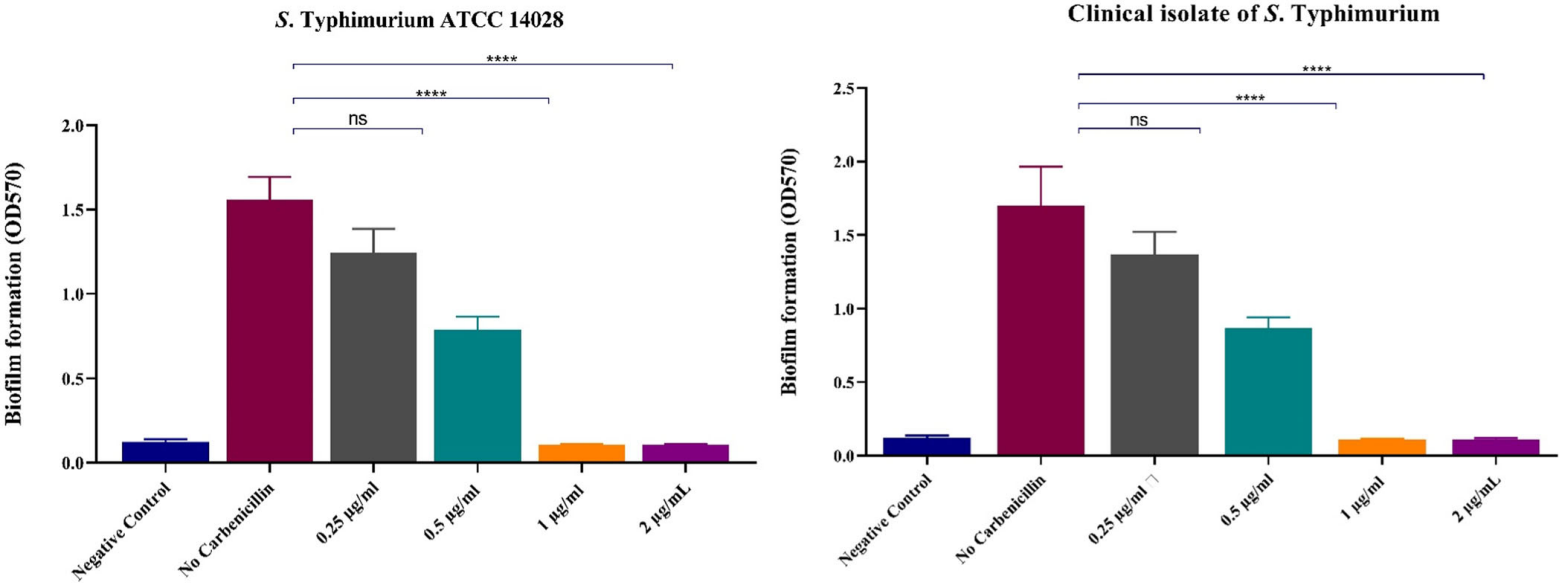
Biofilm formation (A570 nm) of *S*. Typhimurium ATCC and clinical isolate in the presence of different concentrations of Carbenicillin.

**Figure 6:**
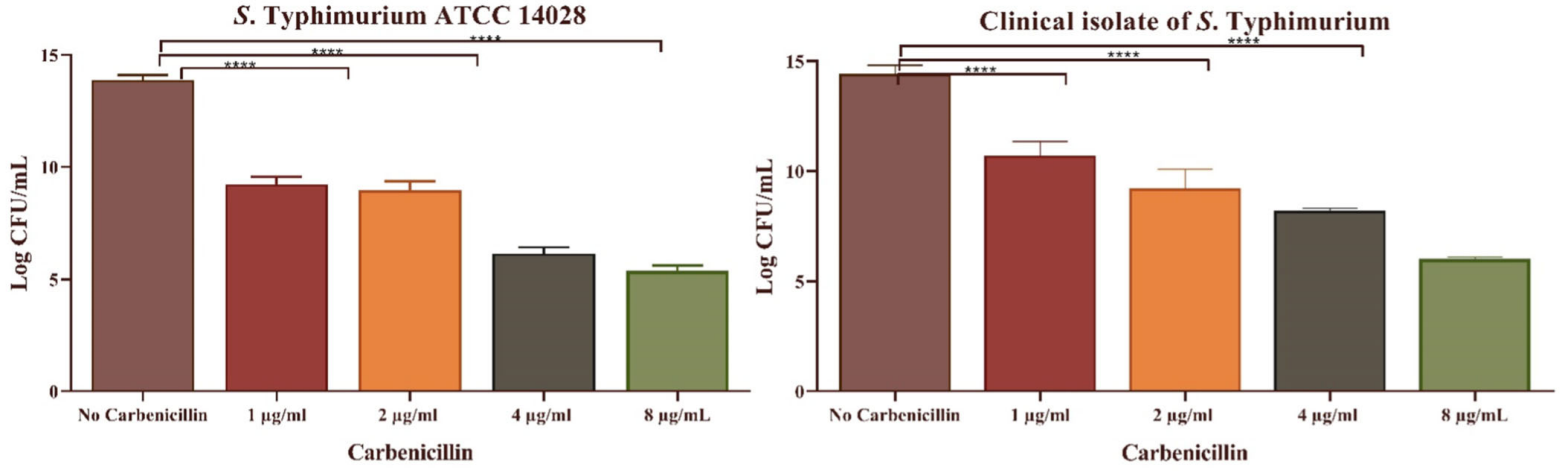
Logarithmic reduction of *S*. Typhimurium ATCC and clinical isolate in the different concentrations of Carbenicillin.

The MBIC of carbenicillin is illustrated in Figure 5. An MBIC of 1 μg/mL was found to significantly suppress biofilm formation in both tested isolates (*p* < 0.0001). The MBEC values are also shown in Figure 6. A carbenicillin concentration of 4 μg/mL resulted in a reduction of over six logs in bacterial counts for both isolates (*p* < 0.0001), establishing this concentration as the MBEC for carbenicillin.

### 3.5 Light Microscopy and SEM Observations

**Figure 7:**
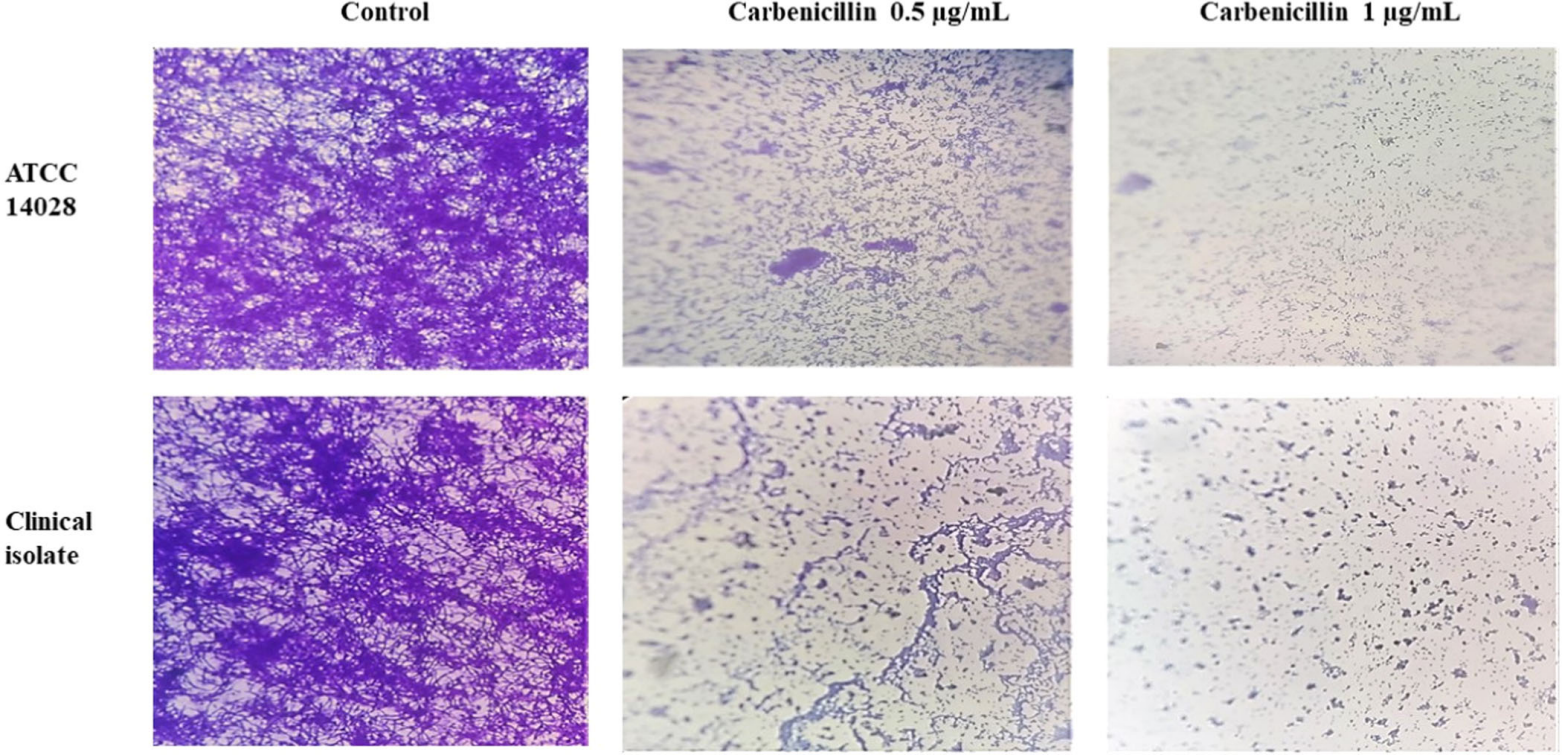
Inhibitory effect of Carbenicillin on the biofilm formation of *S*. Typhimurium ATCC and clinical isolate, evaluated using light microscopy (400x magnification).

Biofilm formation by *S*. Typhimurium isolates was analyzed using light microscopy at ×400 magnification (Figure 7). Treatment with 0.5 μg/mL carbenicillin led to a noticeable reduction in biofilm formation on coverslips, while a concentration of 1 μg/mL effectively inhibited biofilm development in both isolates.

**Figure 8:**
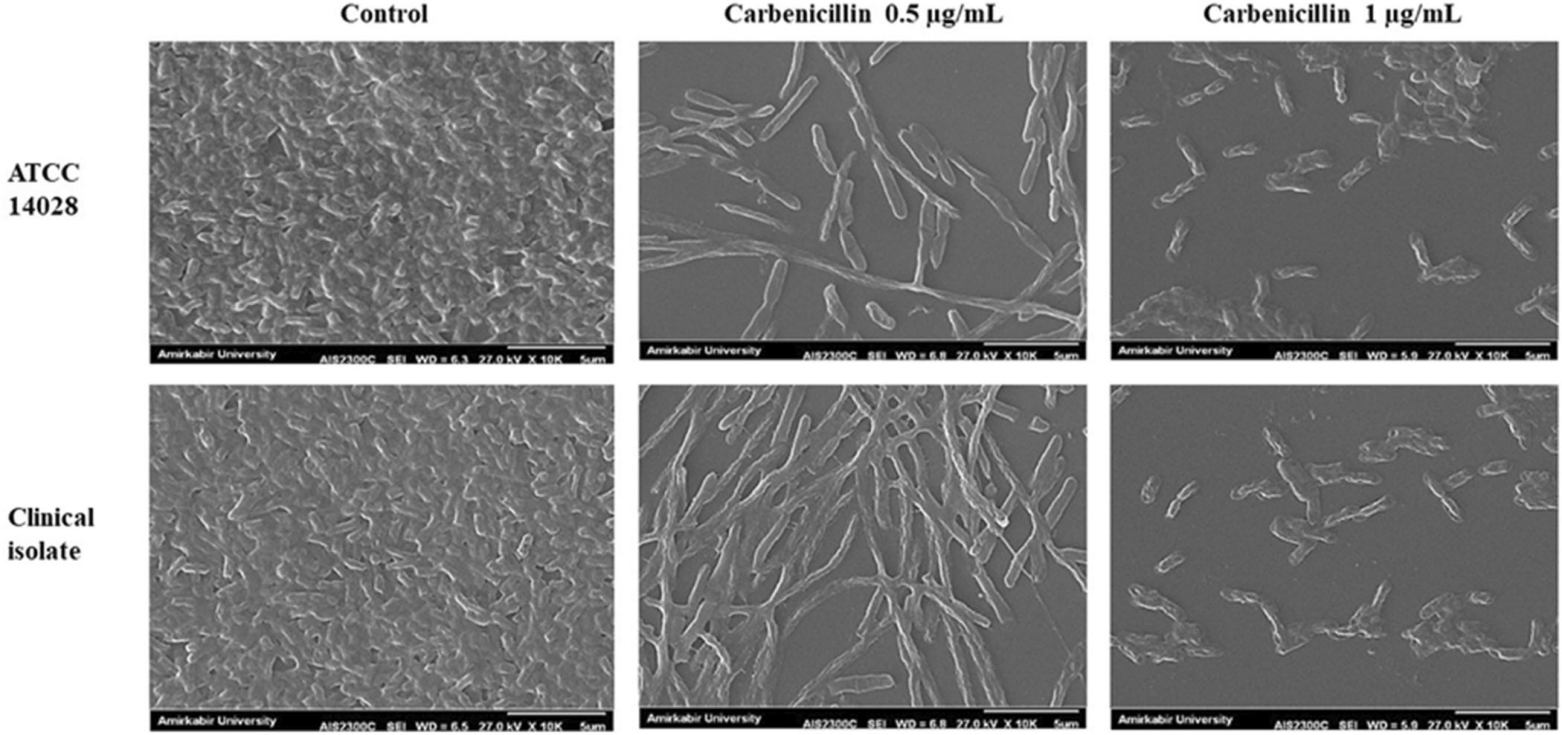
Inhibitory effect of Carbenicillin on the biofilm formation of *S*. Typhimurium ATCC and clinical isolate assessed by scanning electron microscopy at 10000× magnifications.

Scanning electron microscopy (SEM) observations at ×10,000 magnification are presented in Figure 8. At 0.5 μg/mL carbenicillin, a reduction in bacterial counts and exopolysaccharide matrix was observed, accompanied by morphological changes, including elongated and irregular bacterial shapes within the treated biofilms. At 1 μg/mL carbenicillin, a substantial decrease in bacterial numbers, exopolysaccharide matrix, and evidence of bacterial cell destruction were noted.

### 3.6 Curli Production on Congo Red Agar Plates

**Figure 9:**
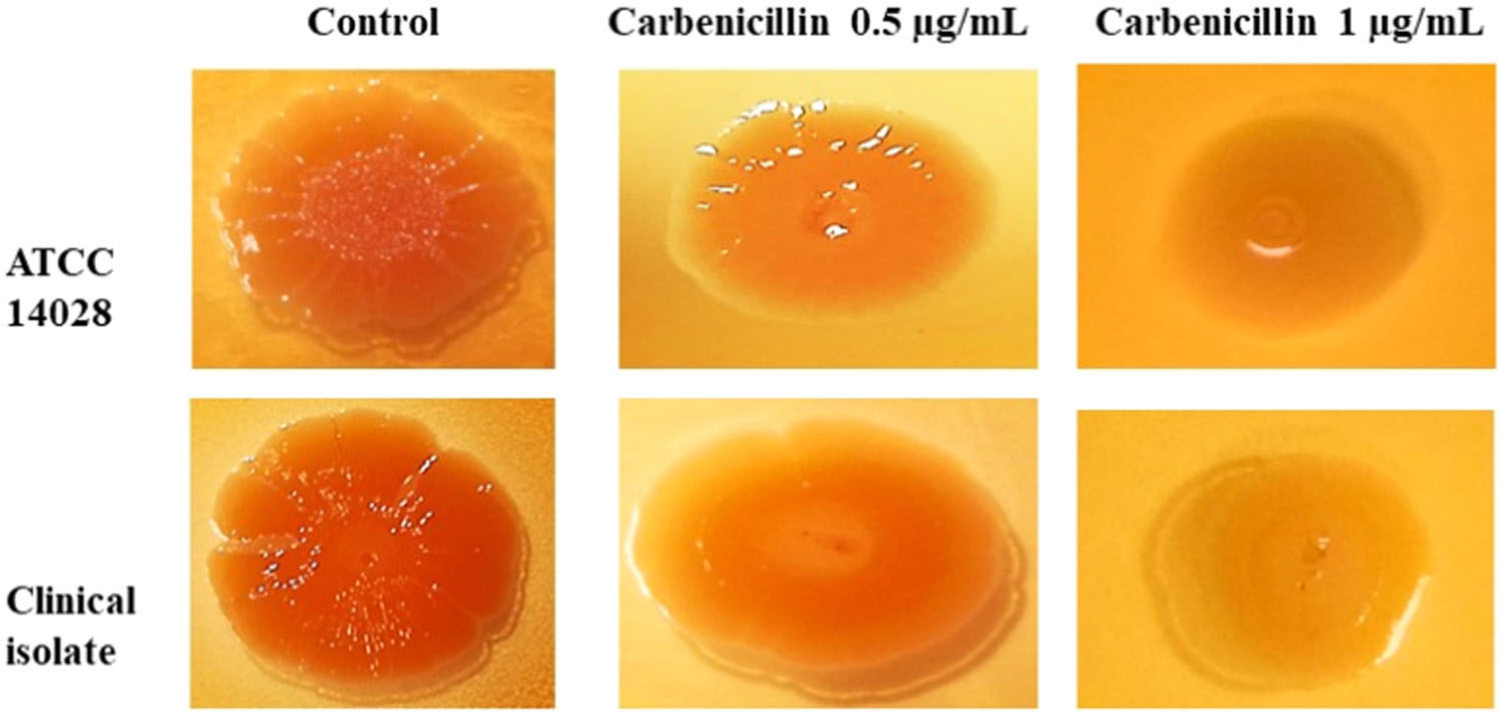
Congo red agar assay experiment on *S*. Typhimurium ATCC and clinical isolate in the different concentrations of Carbenicillin.

The biofilm morphology of control and carbenicillin‐treated *S*. Typhimurium isolates was compared using Congo Red agar plates. In the control condition, isolates formed colonies with pronounced wrinkles and a darker hue, indicative of robust biofilm production. On Congo Red agar supplemented with 0.5 μg/mL carbenicillin, colonies displayed smoother centers and less pronounced wrinkles. On Congo Red agar containing 1 μg/mL carbenicillin, colonies appeared smooth, moist, and largely devoid of wrinkles, suggesting a significant reduction or complete inhibition of biofilm formation (Figure 9).

## 4 Discussion

In *S*. Typhimurium, the transcription factor CsgD plays a pivotal role in biofilm formation by regulating the synthesis of curli fimbriae and cellulose, the primary constituents of the biofilm matrix (Kolypetri et al. 2023).

Drug repurposing, the process of identifying novel therapeutic applications for existing medications, offers a cost‐efficient approach to antimicrobial development (Yang et al. 2019). In the realm of biofilm inhibition, numerous studies have utilized computational screening methods to repurpose drugs targeting biofilm regulatory pathways or quorum‐sensing systems. For instance, D'Angelo, et al. employed virtual screening to identify compounds that inhibit the transcriptional regulator PqsR, a critical component of the quorum‐sensing system in *Pseudomonas aeruginosa* (Adnan et al. 2020). Research reported by Gajdács and Spengler demonstrated that nonantibiotic agents like aspirin, EDTA, curcumin liposomes, and even anticancer compounds can serve as quorum sensing inhibitors and antibiofilm agents in both gram‐negative and gram‐positive pathogens (Li et al. 2024). Given the pivotal role of CsgD in regulating *S*. Typhimurium biofilm formation, this study adopted a drug repurposing strategy, screening FDA‐approved antibiotics to identify inhibitors of CsgD. Our in‐silico screening and experimental validation revealed that carbenicillin, a β‐lactam antibiotic (Grewal et al. 2025), acts as a potent CsgD inhibitor, effectively suppressing biofilm formation.

The *csgD* gene encodes a critical transcription factor that coordinates the expression of structural components such as curli fimbriae and cellulose, the major constituents of the extracellular biofilm matrix (Kolypetri et al. 2023). Genetic studies have shown that disruption or mutation of *csgD* results in a marked decrease in biofilm biomass (Khan et al. 2021). Moreover, such mutants usually display altered colony morphologies, reduced adherence to abiotic surfaces, and impaired resistance to environmental stresses—a phenotype consistent with the loss of curli and cellulose production (Khan et al. 2021; Priya et al. 2015; Zhang et al. 2021).

This study highlights the effectiveness of carbenicillin in suppressing *S*. Typhimurium biofilm formation by targeting the CsgD protein, a critical regulator of curli fimbriae and cellulose synthesis. Experimental assays determined the MIC and MBC of carbenicillin against *S*. Typhimurium at 8 and 32 μg/mL, respectively. In contrast, the MBIC and MBEC were markedly lower, at 1 and 4 μg/mL, respectively, suggesting that carbenicillin disrupts biofilm development at concentrations below those required to inhibit planktonic cell growth. This difference in susceptibility indicates that biofilm‐associated bacteria exhibit distinct response profiles compared to their planktonic counterparts, likely attributable to carbenicillin's interference with CsgD‐mediated biofilm regulation, beyond its conventional bactericidal activity. In a previous study (Narimisa et al. 2024a; 2024b), we utilized a drug repurposing strategy to target the Lon protease, which modulates *S*. Typhimurium biofilm formation through mechanisms including regulation of CsgD and toxin‐antitoxin systems. That study revealed that nafcillin, a Lon protease inhibitor, exhibited a MBIC lower than its MIC for planktonic growth, indicating enhanced efficacy in preventing biofilm formation. These findings parallel the current study's results with carbenicillin, suggesting that targeting regulatory proteins like CsgD or Lon protease may preferentially disrupt biofilm‐specific pathways. The lower MBIC relative to MIC in both studies underscores the potential of repurposed antibiotics to address biofilm‐associated infections through mechanisms distinct from their effects on planktonic bacteria.

Although carbenicillin is classically known for its β‐lactam activity against cell wall synthesis via penicillin‐binding proteins (PBPs) (Thoppil et al. 2008), recent data suggest that it may also possess antibiofilm properties. Studies on burn wound infections have further highlighted carbenicillin's concentration‐dependent bactericidal effects against biofilms formed by pathogens such as *P. aeruginosa*, *Staphylococcus aureus*, and *Escherichia coli* (Thamizhchelvan et al. 2024). Notably, carbenicillin's anti‐biofilm efficacy was enhanced when combined with adjunctive agents, such as maresin‐like pro‐resolving mediators, which amplified its ability to disrupt biofilm viability (Thamizhchelvan et al. 2024; Dusane et al. 2015). In contrast, our study uniquely demonstrates carbenicillin's specific interaction with the CsgD protein in *S*. Typhimurium, suggesting a novel mechanism beyond its bactericidal properties.

This study revealed that carbenicillin treatment at 1 μg/mL significantly altered *S*. Typhimurium colony morphology on Congo red agar, resulting in smooth, moist colonies indicative of suppressed curli fimbriae production. Azam et al. (Azam and Khan 2022), conducted Congo red agar assays on *E. coli* K12 with *csgD* knockdown mutants, observing whitish‐yellow colonies that reflected diminished curli production, in contrast to the red colonies of control cells with robust curli synthesis. Similarly, Wen et al. (Wen et al. 2017). reported that *csgD* deletion in *S*. Typhimurium markedly reduced the red, dry, and rough (rdar) morphotype, yielding white, smooth colonies on Congo red agar, consistent with impaired biofilm formation. In our study, carbenicillin‐induced smooth colony phenotype at 1 μg/mL parallels the reduced curli production observed in these *csgD* mutant studies, reinforcing csgD's critical role in biofilm matrix assembly.

In a prior study, Field Emission Scanning Electron Microscopy (FESEM) imaging revealed that treatment of bacterial biofilms with nafcillin led to significant morphological alterations, including elongation and irregular deformation of bacterial cells within the biofilm matrix (Narimisa et al. 2024a). These structural changes were associated with compromised membrane integrity and potential interference with cell division processes (Malanovic and Lohner 2016). In the present study, similar morphological distortions were observed upon treatment of *S*. Typhimurium biofilms with carbenicillin. At concentration of 0.5 μg/mL, carbenicillin induced notable elongation and irregularity in bacterial cell shape within the biofilm. However, at higher concentrations, carbenicillin completely inhibited biofilm formation and led to bacterial cell destruction.

## 5 Conclusion

This study demonstrates that carbenicillin effectively targets the CsgD protein, disrupting *S*. Typhimurium biofilm formation through direct molecular interactions and potent anti‐biofilm activity. These findings provide a foundation for developing targeted anti‐biofilm therapies, with implications for mitigating the public health burden of *S*. Typhimurium infections. Further mechanistic studies and in vivo validation are warranted to fully explore the therapeutic utility of carbenicillin in biofilm‐associated infections.

## Author Contributions

**Negar Narimisa:** conceptualization, investigation, methodology, software, formal analysis, data curation, visualization, writing – original draft, writing – review and editing. **Amin Khoshbayan:** methodology, investigation, data curation, writing – review and editing. **Faramarz Masjedian Jazi:** resources, supervision, validation, writing – review and editing. **Shabnam Razavi:** resources, supervision, validation, project administration, funding acquisition, writing – review and editing.

## Ethics Statement

The authors have nothing to report.

## Consent

The authors have nothing to report.

## Conflicts of Interest

The authors declare no conflicts of interest.

## Data Availability

The authors have nothing to report.
